# Recent Progress in Obstetrics and Gynæcology

**Published:** 1877-09

**Authors:** S. Howe


### Recent Progress in Obstetrics and Gynæcology.

By S. Howe, M. D.

Obstetrics.—*Is the Fœtus in Utero affected by Medicine which is given the Mother?* *—In the New York Obstetrical Society Dr. Mattison reported a case of puerperal convulsions. The patient was treated with morphia, and was under its influence for about two hours; the morphia was given subcutaneously; the amount was about one and a half grains. The child was born asphyxiated, and shortly after had some convulsions, but finally recovered. An interesting discussion followed the report of the case, the opinions of Zweigel and Fehling being quoted. (Zweigel had found chloroform, after it had been given for some hours to the mother, in the urine of a new-born child. Dr. Fehling's experiment was as follows: A guinea-pig which was about to bear young had injected into its external jugular vein of the left side a large amount of curare; it was kept alive for some hours by artificial respiration. The abdomen was then opened, and the young guinea-pigs were found in a lively condition, unaffected by the drug.)

Dr. Barker opened the discussion by saying that he did not agree with Dr. Mattison, but thought that convulsions in a fœtus might take place due to opium poisoning. He cited cases in animals where opium poisoning was followed by convulsions, and said that

* American Journal of Obstetrics, March.

in those savage races in which the brain is less developed than in the rest of mankind convulsions do occur after toxic doses of opium. The possibility of a poison passing from the blood of the mother to that of the fœtus is shown in cases of scarlatina, variola, and syphilis. That medicine does not pass from mother to fœtus the frequent unsuccessful or negative results of antisyphlitic treatment spoke very strongly. He mentioned a case of a syphilitic child born from a mother free from the disease, which had been latent in the father for a long time. At the next pregnancy he had the woman for six months under the mercurial cure, and, nevertheless, five days after the birth the child began to develop well-marked syphilitic symptoms. But what spoke strongest against the passage of drugs from the mother to the child was the effect of anæsthetics. Dr. Barker said that during the last twenty-five years he had administered chloroform over one thousand times and had never seen a case where the death of the child could be attributed to chloroform; that he had kept women from eight to twelve hours under the influence, and in one case had given three and a half pounds. He thought that opium acted nearly in the same way. Formerly he had been very careful in giving opium to a pregnant woman, but after his experience in the following case he had always given it without fear. The case was one of puerperal convulsions; the fœtus was thought dead, and morphia was used very freely. The patient was a long time under the influence of the drug, when a living child was born. In conclusion, he said that he considered that the fœtus in utero was not affected by medicine, and more especially narcotics, when administered to the mother.

After this, several other physicians concurred in the same opinion. Dr. Gillette spoke against the views expressed by Dr. Barker and others, and was of the opinion that morphia would affect the child through the mother. He cited the case of a pregnant woman with some valvular leision of the heart, who during her labor was under the influence of morphia; when the child was born it was very much asphyxiated, and acted, Dr. Gillette said, as if it had been poisoned with opium. (Perhaps the asphyxia was due as much to the valvular lesion of the mother's heart and the imperfect oxygenation of the blood as to the morphia.) He mentioned six other cases where morphia had been given and the children at birth were all very much asphyxiated; pulse slower than normal; pupils contracted. In all these cases the labors were normal and the pains not weak. More important, however, are the two following cases which he cited: Two women during labor had one-thirtieth and one forty-eighth of a grain of sulphate of atropia. In the first case the child was born in thirty minutes after the administration of the drug, and the child was unaffected by it. In the second case the birth did not take place for three hours, and the pupils of the child were very much dilated and did not contract in a strong light.

Dr. Jacobi thought that the amount of morphia necessary to produce sleep, or relieve pain in a woman would be so diluted in the mother's blood that when it came in contact with the fœtal circulation it would be harmless.

Dr. Thomas agreed with Dr. Gillette, and thought that opium did produce a marked effect on the fœtus. In two cases he had observed that after the administration of morphia the fœtal pulse fell in one case from 141 to 119, and in the other from 133 to 118.

Dr. Lusk * has a paper on this subject. After trying morphia in labor in his own cases, and from notes of cases of a friend, Dr. Beckwith, of the Nursing and Children's Hospital, he comes to the conclusion that morphia in no way affects the child. In the same Journal Dr. Lamadrid reports a very interesting case of death of the fœtus, which he attributes to doses of morphia and chloral given two weeks before for neuralgia in a pregnant woman; that after the administration of the drug the motions of the child ceased, and when the child was born it appeared as if it had been dead two weeks.

The evidence seems on the whole to point to the opinions expressed by Drs. Barker, Lusk, and others, that under ordinary circumstances medicines given to the mother produce no effect on the fœtus, and only in rare cases can they be said to be hurtful. Of course, before the matter is settled many more careful observations are necessary.

*Face Presentations.*—Dr. W. Groner † recommends, in cases where the face presents, that the attempt to change this unfortunate position into the normal occipital one should be made. The change can be effected by external manipulation in most cases, and if not by external alone, by combined. The operation he calls Schantz's. He gives three cases, in two of which he was able to bring about this change by external manipulation, and in the other by the combined method, external and internal. Korman ‡ reports a case where he accomplished this by the combined method. J. R. Humphrey, M. D., reports § a case of face presentation which he succeeded in changing into the ordinary occipital one by placing the patient in the so-called knee-elbow position.

E. L. Partridge ‖ reports two cases of face presentation, in both of which he was able to bring on the desired change from the face to the ordinary occipital position by the combined method of external and internal manipulation. The operation was performed in the following manner: The os uteri nearly or wholly dilated and the membranes still intact, the patient was placed in a position which was easiest for the operator. The left hand was passed into the vagina, and two fingers pushed through the os; the membranes

---

* American Journal of Obstetrics, July.
† Archiv. fur Gynækologie. volume xi., part 2, page 235.
‡ Deutsche medizinische Wochenschrift, No. 5.
§ American Journal of the Medical Sciences, January, 1877.
‖ New York Medical Journal, March, 1877.

were then ruptured, but as the vagina was stopped by the hand the liquor amnii was prevented from escaping. Two fingers were hooked over the occiput, and it was dragged down into the cavity of the pelvis; the right hand was used to press on the fundus of the uterus and hold the child in the new position. The occiput was held down as long as possible, and the external pressure was kept up until the head was well engaged. The rest of the labor was normal.

*Influence of Posture on Women.*—J. H. Aveling, M. D.,* has written a long article on the influence of position on women in regard to menstruation, ovulation, pregnancy, labor, etc. He says that posture has a marked effect on ovulation and the anomalies of menstruation; that women who are in the habit of sitting or working in one position are more liable to hyperæmia of the ovaries; that abdominal and tubal pregnancy is sometimes due to the effects of position; and that some of the various troubles of menstruation are influenced by the position which women keep.

The ordinary position of the woman during coitus is often a cause of sterility, and with a change of posture pregnancy will sometimes occur. During pregnancy the posture of the woman may cause change in position in the os uteri and uterus itself, and that toward the end of pregnancy unfortunate position or movement may cause the labor to begin. That position has a marked influence over labor is well known, and during the child-bed sickness the position on the back, if kept constantly, will often cause trouble. He recommends, therefore, during child-bed, change from the back to the side often; that at meal time and when the child is being nursed it is advisable for the woman to sit up in bed; and to prevent the lochia from remaining in the vagina and also to aid in thoroughly emptying the bladder the woman should get on her hands and knees for a few moments.

*The Passage of Salicylic Acid and Iodide of Potash from the Mother into the Liquor Amnii.*—Max Ruerge† has an article on this subject of some length, and as the result of trying many experiments he comes to the conclusion that, after giving a pregnant woman salicylic acid in twenty-five grain doses from ten to fifteen days, or about half as much iodide of potash, a very slight trace of these substances can be detected in the liquor amnii; but he considers it most probable that the passage is not direct from the woman's blood to the liquor amnii, but that it first goes into the fœtus, and from the fœtus probable through the urine of the same into the liquor amnii. The amount discovered is always very slight, and it is only with the most delicate tests that it can be detected.

*New Forceps.*—M. Talnier‡ has invented a new pair of forceps which have a third curve. The handles are curved like a letter S:

---

* The Obstetrical Journal of Great Britain and Ireland, from January to April.
† Centralblatt for Gynaekologie, No. 5.
‡ Annales de Gynecologie, March, 1877.

the upper segment represents the blades, the lower the handles. In the lower ends of the blades traction shafts are fitted, possessing a curve corresponding to that of the handles. A cross-bar unites them at their lower ends, and is the point from which traction is made. There is a screw on the handles by which the blades are tightened over the fœtal head. Each blade is applied with its traction shaft adjusted. M. Talnier asserts that by means of these forceps the traction can be made always in the direction of the axis of the pelvis, whatever may be the position of the fœtal head. He says that it is a real instrument of traction, and not a lever like the ordinary forceps. It allows the fœtal head to follow the curve of the pelvis with freedom.

*Instrumental Delivery without the Knowledge of the Patient.*—Dr. James Braithwaite * describes a new forceps with which he has delivered thirty-eight times in three hundred and eighty-four labors, and thirty-seven children were born alive. Often he has been able to use them without the knowledge of the woman. The instrument is much lighter than the ordinary forceps, and has this peculiarity, that it is introduced as one blade. One of the blades fits into the other, and is held in position by a cap which passes over the ends of the handles. When the instrument is introduced the cap is removed, and the two blades are twisted into position. The blades are introduced into the hollow of the sacrum. The blades are then locked in the ordinary way and the fœtus extracted.—*Boston Med. Journal.*

* The Obstetrical Journal of Great Britain and Ireland.

